# Editorial: Drug Survival: Treatment of Rheumatic Diseases in the Biologic Era

**DOI:** 10.3389/fmed.2022.858817

**Published:** 2022-02-18

**Authors:** Lorenzo Di Sanzo, Rossana Scrivo, Enrique Roberto Soriano, Gustavo Citera, Eduardo Mysler, James Cheng-Chung Wei, Mario Humberto Cardiel Ríos

**Affiliations:** ^1^Rheumatology Unit, Department of Clinical Internal, Anesthesiological and Cardiovascular Sciences, Sapienza University of Rome, Rome, Italy; ^2^Italian Hospital of Buenos Aires, Buenos Aires, Argentina; ^3^Instituto Nacional de Rehabilitación Psicofísica, Buenos Aires, Argentina; ^4^Independent Researcher, Buenos Aires, Argentina; ^5^Department of Internal Medicine, Chung Shan Medical University Hospital, Taichung, Taiwan; ^6^Centro de Investigación Clínica de Morelia, Morelia, Mexico

**Keywords:** treatment survival, persistence, biologic agents, inflammatory arthropathies, arthritis

The development of personalized medicine to improve the outcome of patients with chronic inflammatory rheumatic diseases is yet far to come. Personalized treatment strategies are supposed to foster the possibility of addressing the right therapies to the right patients (1), identifying, in the first place, subgroups of patients for whom a different treatment strategy would be beneficial. Biologic agents targeting various molecules involved in the pathogenesis of many inflammatory diseases are meant to respond to this need. However, despite representing a major advance in the treatment of these conditions, a significant proportion of patients does not achieve the identified target of remission or low disease activity (2–4). In the absence of biomarkers that may be predictive of response to any of these drugs, treatment survival is considered a surrogate outcome measure of both effectiveness and safety, especially in the real-world setting, which allows a prolonged observation of patients compared to randomized clinical trials. Also, prolonged treatment survival may help recognize the characteristics of patients associated with a better clinical outcome, thus supporting the decision on drug choice. This is a critical issue in an era that sees an expanding landscape of treatment opportunities for many patients with inflammatory diseases.

This Resaerch Topic of Frontiers in Medicine is intended to capture evidence of biologic agent survival in clinical practice, collecting a series of papers on patients with chronic inflammatory arthropathies and vasculitis. Two of these papers involve patients with rheumatoid arthritis (RA) and the role of concomitant conventional synthetic antirheumatic drugs (csDMARDs), specifically methotrexate (MTX), in treatment survival. In particular, in a Spanish study, patients starting their first anti-TNF agent were investigated to ascertain whether seropositive status could affect treatment survival (Hernández-Breijo et al.). Interestingly, this was not the case, unless patients receiving concomitant MTX were seropositive. Indeed, MTX is known to decrease B cell activity and hence the immunogenicity associated with anti-TNF drugs (5). However, in the other RA study, no significant differences in the retention rate among patients receiving the anti-TNF agent certolizumab pegol (CZP) with and without MTX and patients receiving the high vs. low MTX doses were observed (Nozaki et al.). Polyethylene glycol contained in the CZP molecule serves to increase the half-life of a protein *in vivo*, which may, at least in part, explain these results. It is important to underline that the study was set in Japanese patients in a non-randomized fashion, hence the findings cannot be generalized.

Asian patients were analyzed in another study focusing on ankylosing spondylitis (AS) (Kim et al.). In this paper, the retention rate of different anti-TNF agents was compared using data from the Korean College of Rheumatology Biologics registry. Golimumab demonstrated to have higher persistence, but this result might have been affected by the local health insurance policy. A comprehensive view of drug survival was obtained in patients with RA, AS, and psoriatic arthritis (PsA) stratified according to the treatment line in Australia, a country with relatively strict Pharmaceutical Benefits Scheme criteria for access to biologic agents (Bhushan et al.). Upon the examination of the results, the first point was the lack of association of biologic agent failure with concomitant use of csDMARDs, differently from other studies. Also, similar persistence rates of the first, second, and third-line drugs in RA, AS and PsA were found, with the retention rate of the first drug similar to previous years, despite the current greater availability of drugs with different mechanisms of action.

This Resaerch Topic of Frontiers in Medicine also gives space to iguratimod, a non-biologic small molecule with proven effectiveness and safety in patients with RA from China and Japan. In the published paper, iguratimod was tested in patients with active spondyloarthritis, showing a significant improvement in signs and symptoms as well as the quality of life after 24 weeks against patients randomized to placebo (Li et al.).

Only one paper in this issue investigates the clinical outcome in patients with vasculitis. This was an Italian study on 23 patients affected by Takayasu arteritis (TAK) resistant to conventional treatment, either already treated with infliximab (IFX)-originator (switch group) or never treated with IFX (naïve group), who received CT-P13 IFX biosimilar (Campochiaro et al.). CT-P13 retention rate was 90.9% at month 6 and 90.4% at month 12. Indeed, CT-P13 showed satisfactory results both in clinical manifestations assessed by clinimetric scores and vascular inflammation assessed by imaging techniques, including magnetic resonance angiography and (18F)-FDG-PET. In addition, patients were able to reduce the mean daily dose of glucocorticoids at 6 months, and this achievement was maintained at 12 months. The safety profile was excellent, further supporting the use of IFX in refractory TAK.

Finally, interesting insights for reflection on unmet needs in the management of patients with inflammatory rheumatic diseases come from Romanian authors, who face the problem of residual pain (RP) in their mini-review (Berghea et al.). The possible causes of RP are explored (chronic nociceptive, neuropathic, nociplastic pain), supporting the possibility that it may be part of the post-remission syndrome (PRS), a condition described after obtaining remission. This hypothesis raises several questions, leading to investigate whether PRS pain may be a common outcome rather than an individual response in patients not properly treated, or to examine the opportunity to change or integrate treatment in patients with RP, thus potentially influencing treatment survival.

We hope this special issue of *Frontiers in Medicine* meets the interest of the readers, tickling further research about drivers of drug selection in inflammatory rheumatic diseases.

## Author Contributions

LDS wrote sections of the manuscript. RS contributed to conception of the manuscript, wrote the first draft of the manuscript, and reviewed the final version. All authors approved the submitted version.

## Conflict of Interest

The authors declare that the research was conducted in the absence of any commercial or financial relationships that could be construed as a potential conflict of interest.

## Publisher's Note

All claims expressed in this article are solely those of the authors and do not necessarily represent those of their affiliated organizations, or those of the publisher, the editors and the reviewers. Any product that may be evaluated in this article, or claim that may be made by its manufacturer, is not guaranteed or endorsed by the publisher.
